# Computational analysis of affinity dynamics between the variants of SARS-CoV-2 spike protein (RBD) and human ACE-2 receptor

**DOI:** 10.1186/s12985-024-02365-3

**Published:** 2024-04-19

**Authors:** Nishad Sultana, S. N. Nagesha, C. N. Lakshminarayana Reddy, B. N. Ramesh, S. Shyamalamma, K. S. Shashidhara, K. M. Satish, C. Pradeep, G. D Vidyadhar

**Affiliations:** 1 Department of Plant Biotechnology, University of Agricultural Sciences, GKVK, Bangalore, 560 065 India; 2 Department of Plant Biotechnology, College of Agriculture, Hassan, UAS, Bangalore, 573 225 India; 3 Department of Plant Pathology, University of Agricultural Sciences, GKVK, Bangalore, 560 065 India; 4 ICAR-PHT, UAS, GKVK, Bangalore, 560 065 India; 5 Department of Genetics and Plant Breeding, College of Agriculture, Hassan, UAS, Bangalore, 573 225 India; 6 Department Biotechnology, KSNUAHS, Shivamogga, 577 412 India

**Keywords:** SARS-CoV-2, h-ACE2, Variants of concern (VOCs), HADDOCK, trRosetta

## Abstract

The novel coronavirus SARS-CoV-2 resulted in a significant worldwide health emergency known as the COVID-19 pandemic. This crisis has been marked by the widespread of various variants, with certain ones causing notable apprehension. In this study, we harnessed computational techniques to scrutinize these Variants of Concern (VOCs), including various Omicron subvariants. Our approach involved the use of protein structure prediction algorithms and molecular docking techniques, we have investigated the effects of mutations within the Receptor Binding Domain (RBD) of SARS-CoV-2 and how these mutations influence its interactions with the human angiotensin-converting enzyme 2 (hACE-2) receptor. Further we have predicted the structural alterations in the RBD of naturally occurring SARS-CoV-2 variants using the tr-Rosetta algorithm. Subsequent docking and binding analysis employing HADDOCK and PRODIGY illuminated crucial interactions occurring at the Receptor-Binding Motif (RBM). Our findings revealed a hierarchy of increased binding affinity between the human ACE2 receptor and the various RBDs, in the order of wild type (Wuhan-strain) < Beta < Alpha < Gamma < Omicron-B.1.1.529 < Delta < Omicron-BA.2.12.1 < Omicron-BA.5.2.1 < Omicron-BA.1.1. Notably, Omicron-BA.1.1 demonstrated the highest binding affinity of -17.4 kcal mol^−1^ to the hACE2 receptor when compared to all the mutant complexes. Additionally, our examination indicated that mutations occurring in active residues of the Receptor Binding Domain (RBD) consistently improved the binding affinity and intermolecular interactions in all mutant complexes. Analysis of the differences among variants has laid a foundation for the structure-based drug design targeting the RBD region of SARS-CoV-2.

**Supplementary Information:**

The online version contains supplementary material available at 10.1186/s12985-024-02365-3.

## Introduction

COVID-19, caused by the SARS-CoV-2 virus, emerged in Wuhan, China, in December 2019 [1], evolving into a global pandemic. Its symptoms vary but commonly include fever, cough, headache, fatigue, breathing difficulties, loss of smell, and loss of taste, typically manifesting one to fourteen days after exposure. As of September 4, 2023, the World Health Organization (WHO) reported 770,563,467 confirmed cases and 6,957,216 fatalities worldwide [2]. SARSCoV-2 has spread to more than 200 nations and territories since the World Health Organization (WHO) first received a report of it in December 2019, [(As of 4 September 2023, daily online worldwide data concerning COVID-19, (https://www.who.int/)]. The virion’s surface features a type I fusion glycoprotein known as the spike glycoprotein, consisting of various trimers with two subunits: S1 and S2. The S1 subunit, located on the viral membrane, encompasses the N-terminal domain (residues 14 − 305) and forms the receptor‐binding domain (RBD) (residues 319 − 541), crucial for replication [3]. The RBD of the spike glycoprotein specifically recognizes the human angiotensin‐converting enzyme receptor (hACE2R) in the respiratory system, facilitating the entry of the virus into the human host cell. The spike glycoprotein of the SARS‐CoV‐2 variant that emerged in Wuhan in 2019 exhibited a binding capacity to hACE2R 10 to 20 times higher than the virus from 2002 [4]. The World Health Organization (WHO) coined the term “Variant of Concern” (VOC) to identify viral variants with mutations in their spike glycoprotein, altering their binding affinity to hACE2R [5]. Notable VOCs include Alpha (B.1.1.7‐lineage), Beta (B.1.351), Gamma (P.1), Delta (B.1.617.2), and the more recent Omicron (B.1.1.529), which emerged in South Africa. Recent literature suggests that the RBD of the Omicron variant (B.1.1.529) has developed a heightened affinity and binding capacity to hACE2R, contributing to increased transmissibility [6]. Studies indicate that Omicron, with 16 mutations on the RBD, is notably more transmissible than previous variants [7]. Consequently, it is imperative to investigate the novel mutations in the Omicron‐RBD variant to understand their interaction with hACE2R. In this investigation, we employed the trRosetta algorithm for *de novo* or ab initio prediction of the 3D structures of COVID-19 receptor-binding sequences. Subsequent refinement of the predicted complexes was carried out using HADDOCK, an information-driven flexible docking approach, along with PRODIGY, a binding affinity descriptor based solely on the structural properties of the protein–protein complex between RBD mutations and the ACE-2 receptor. Our study focused on predicting the 3D structures of various naturally occurring COVID-19 variants and comparing them to the structures obtained from an experimental PDB structure and the original Wuhan strain. This comparative analysis shed light on variations in the RBD and its interactions with the ACE-2 receptor, particularly concerning infiltration and infectivity, pinpointing residues within the Receptor-Binding Motif (RBM). Additionally, we utilized trRosetta to predict potential mutations at specific conserved RBM residues across different naturally occurring variants. The study extended to forecasting the interactions of these mutations with the ACE-2 receptor using HADDOCK and PRODIGY. The methodology introduced in this study forms a comprehensive pipeline, offering a valuable approach for future investigations into COVID-19 and serving as a guide for developing strategies to combat viral diseases, particularly through the design of specific subunit vaccines.

## Materials and methods

### Retrieval of spike glycoprotein(S) amino acid sequence from NCBI SARS-CoV-2 data hub

The amino acid sequences of the S proteins analysed in this study were collected at quarterly intervals, covering the period from December 1, 2019, to March 1, 2023. These sequences were obtained from the NCBI Viruses SARS-CoV-2 Data Hub (http://www.ncbi.nlm.nih.gov), and each sequence was associated with its respective accession number, further the RBD region i.e., from 319 to 541 amino acid sequence was retained from all the spike glycoprotein sequences for further study using Biopython (Table S6, Supplementary Data). Till date, the 6 major variants of SARS-CoV2 i.e., Original Wuhan strain, Alpha, Beta, Gamma, Delta and Omicron (https://www.who.int/activities/tracking-SARS-CoV-2-variants) from the top 5 majorly affected countries with COVID-19 which includes USA, India, France, Germany and Brazil (https://covid19.who.int/table) were considered for this study.

### Multiple sequence alignment of the receptor binding domain of spike glycoprotein amino acid sequences

The spike glycoprotein sequences were sourced from the NCBI virus SARS-CoV-2 Data Hub. Subsequently, utilizing Biopython, the receptor-binding domain (RBD) segment spanning residues 319 to 541 was separated [8]. This process facilitated the creation of distinct FASTA files containing the sequences corresponding to each variant as observed within individual countries. The RBD region of the spike glycoprotein underwent multiple sequence alignment using Clustal W within the BioEdit software (version 7.2.5) [9]. Refer to Figure S1 in the Supplementary Data for a visual representation of the alignment results.

### Phylogenetic analysis of RBD region of spike glycoprotein

The phylogenetic analysis of the SARS-CoV-2 RBD region for the top 5 countries was conducted using MEGAX software (MEGA-X Version 11) [10]. This analysis involved multiple comparisons implemented through the neighbour-joining algorithm within MEGA-X. The process included multiple sequence alignments using Clustal W, and the neighbour-joining phylogenies were constructed based on 156 sequences gathered from the NCBI virus SARS-CoV-2 Data Hub. An outgroup, HCoV 229E, was used for reference. The evolutionary history was inferred utilizing the neighbour-joining method, with the percentage of replicate trees indicating the bootstrap support (100 replicates) displayed next to the branches. The evolutionary distances were calculated using the Poisson correction method and are presented in the units of the number of amino-acid substitutions per site. All evolutionary analyses were carried out in MEGA X. The numbers at the nodes represent the bootstrap support from 100 replicates, and the scale bar illustrates the estimated number of substitutions per site. Refer to Figure S2 and S3 in the Supplementary Data for visual representations of the phylogenetic analyses.

### The 3D modelling of RBD of spike glycoprotein to determine the conformational changes due to mutations

The 3D protein models for the Receptor-Binding Domain (RBD) region of the spike glycoprotein of SARS-CoV-2 were constructed using the trRosetta (transform-restrained Rosetta) server for structural modeling [11]. Subsequently, the models generated for naturally occurring variants were superimposed and aligned with the original Wuhan variant using PyMol software [12]. The PyMol software’s “super” command was employed for the alignment process. This command facilitates the superposition of two protein selections, utilizing a sequence-independent structure-based dynamic programming alignment. Unlike the “align” command, “super” incorporates a series of refinement cycles aimed at enhancing the fit by eliminating pairings with high relative variability. This method is particularly advantageous for proteins with low sequence similarity. The output of the “super” command includes a numerical Root-Mean-Square-Deviation (RMSD) value, providing a quantitative measure of the structural differences between the aligned protein structures. This RMSD value serves as an indicator of the level of conformational variation among the models.

### Protein − protein docking of RBD region and ACE2 of human

The study conducted docking analyses between ACE2 and the RBD of various variants, utilizing the “EASY” access level provided by the HADDOCK 2.4 server [13]. The chosen cluster models were retrieved in PDB format for subsequent analysis. The process involved selecting the initial model with the least binding free energy value. Active and passive residues were defined to offer insights into interacting residues, serving as potential constraints during the docking process. The HADDOCK docking methodology, considering intermolecular interactions within a 6.5 Å cut-off, allows flexibility in establishing the protein − protein interface. Consequently, distinct flexible zones were defined for each binding pose. The identification of intermolecular interactions automatically delineated semiflexible residues [14].

### Determination of dissociation constant (KD) and comparison of interactions between mutant RBD and human ACE2 receptor

The binding affinity and dissociation constant (KD) were determined using the PROtein Binding Energy Prediction (PRODIGY), an automated server designed for calculating binding affinities in various biological complexes [15]. Subsequently, the calculated binding affinities for the spike Receptor-Binding Domain (RBD) and hACE2R complexes were explored in more detail using PyMol 2.5.2. PyMol is a molecular visualization software that allows for the analysis and visualization of complex structures. In this context, PyMol was used to examine the interaction profile of the spike RBD-hACE2R complexes, providing a visual representation of the molecular interactions within the complex structures. This analysis aids in understanding the spatial arrangement and key interactions between the spike RBD and hACE2R, contributing to a comprehensive characterization of the binding interfaces.

## Results and discussion

### Mutations in RBD and structure stability of SARS-CoV-2 virus VOCs

**Fig. 1:**
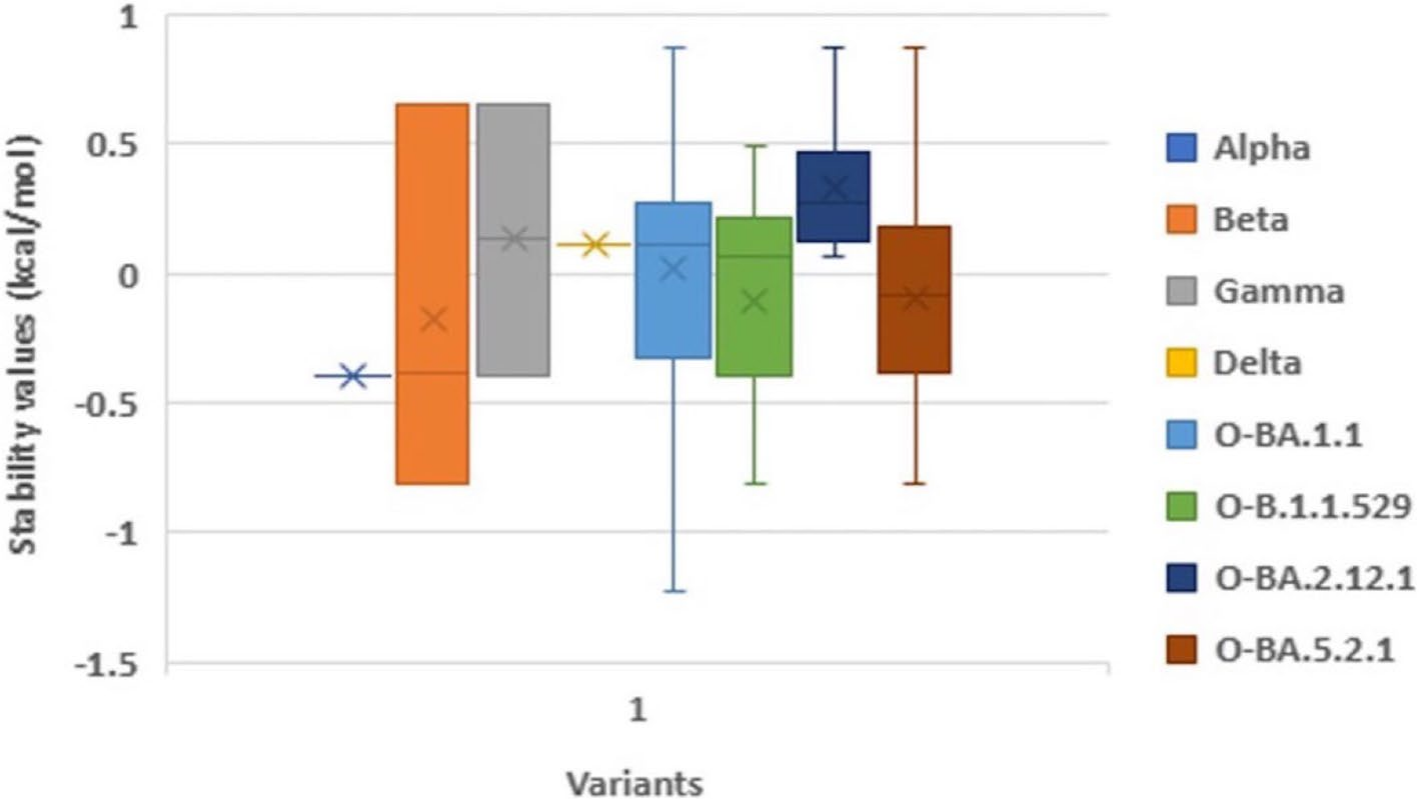
The stability changes of each mutation of different variant of concern and the omicron subvariants and their mean stability values

Prior bioinformatic investigations have highlighted the pivotal role of the spike protein’s Receptor-Binding Domain (RBD) in susceptibility to novel mutations and its critical function in host binding [16]. To comprehensively assess the RBD within the SARS-CoV-2 spike protein, a multiple sequence alignment was employed. This procedure involved the comparison of RBD sequences from SARS-CoV-2 strains originating from the top five nations significantly impacted by the pandemic, as documented by the World Health Organization (WHO), where substantial fatalities were recorded. The reference sequence for this alignment was human SARS-CoV-2 virus reported in Wuhan, China, with accession number YP_009724390.1. The Alpha variant (B.1.1.7), first identified in the United Kingdom, displayed the N501Y mutation within its RBD region. This mutation has raised concerns about potential implications, such as increased viral load, extended viral persistence, and an elevated risk of fatality when compared to the reference sequence [17] (Table S1, Supplementary Data). In South Africa, the Beta variant (B.1.351) presented three distinctive mutations within its RBD region, including K417N, E484K, and N501Y, which have been associated with a notable reduction in antibody neutralization [18] (Table S2, Supplementary Data). Similarly, the Gamma variant (P.1) discovered in Brazil exhibited mutations K417T, E484K, and N501Y in the RBD region, with an estimated 2.6-fold increase in transmissibility [19] (Table S3, Supplementary Data). The Delta variant (B.1.617.2), originating in India, was characterized by L452R, E484Q, and T478K mutations within the RBD, leading to a significant surge in cases and hospitalizations during India’s second wave [19] (Table S4, Supplementary Data). Lastly, the Omicron variant (B.1.1.529) in South Africa presented an array of prominent mutations, including G339D, S371L, S373P, S375F, K417N, N440K, G446S, T478K, E484A, Q498R, G496S, Q498R, N501Y, and Y505H. These mutations have raised concerns regarding the potential for increased reinfection risk compared to other VOCs [20] (Table S5, Supplementary Data). Enhanced virus infectivity can stem from improved host receptor binding stability. The strength of each unique RBD mutation in each variant was assessed (Table 1). Missense mutations within RBDs can impact their binding affinity to hACE2R. Using the DUET webserver, missense mutations were evaluated in the RBD protein and protein stability changes were recorded. The mean ΔΔG values in monomer stability in each full-length RBD-residue position ranged from − 1.229 kcal/mol in G496S to 0.876 kcal/mol in N440K. The results depicted in Fig. 1 highlight specific amino acid (AA) positions that play a significant role in either stabilizing or destabilizing the Receptor-Binding Domain (RBD) of the spike glycoprotein, with corresponding percentages listed in Table 6. Noteworthy glycoprotein mutations, including G339D, S371L, N440K, S477N, T478K, E484A, E484K, Q493R, Q498R, and Y505H, demonstrated pronounced stabilizing effects on the protein structure. The analysis revealed that among the 22 unique AA substitutions, only 47.6% exhibited stabilizing effects, while 52.3% had destabilizing effects on the Spike Protein. A particularly notable observation was the high stabilizing effect exerted by the N440K transformation in all Omicron variants, indicated by a ΔΔG value of 0.876 kcal/mol. Comparisons between RBD mutations in Beta and Gamma (E484K; ΔΔG: 0.654 kcal/mol) and Omicron subvariants (Q483R; ΔΔG: 0.495 kcal/mol) revealed that Beta and Gamma variants showed significant stabilizing effects. Conversely, Delta and B.1.617.2 (L452R) and Beta and Omicron variants (K417N) exhibited notable destabilizing impacts, as indicated by low ΔΔG values when predicting missense mutations’ effects on protein stability (see Fig. 1).

**Table 1:**
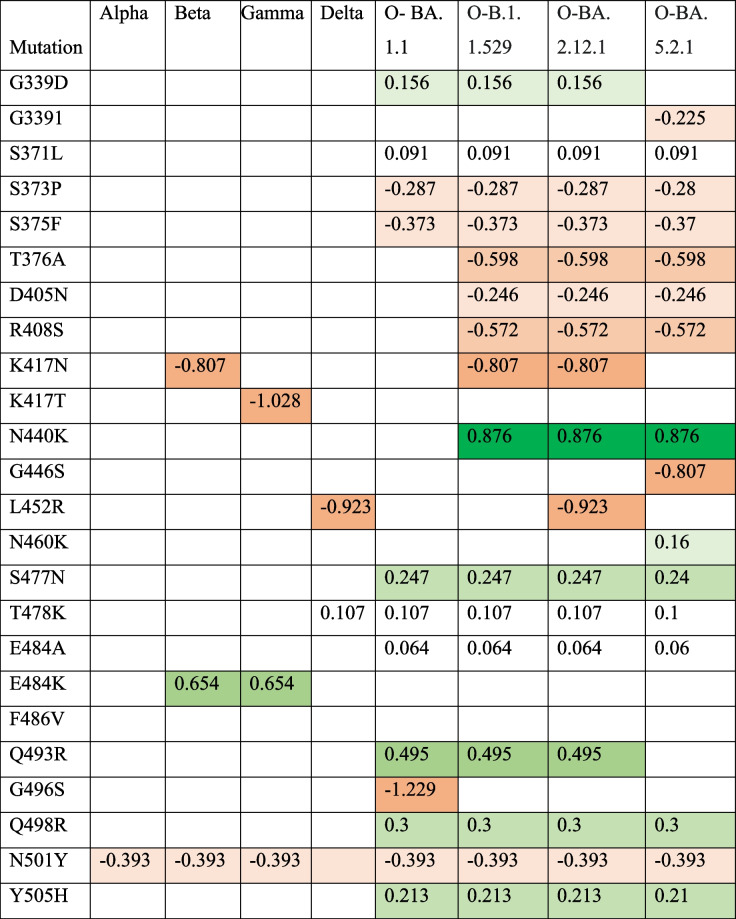
Main mutations in the RBD of Variants of concern and subvariants of the Omicron and their stability values generated from the DUET webserver

Red color destabilizing mutations, Green color stabilizing mutations. All the stability values are presented in kcal/mol

*Abbreviations*: *D* Decreased stability, *De* Destabilizing, *In* Increased stability, *O* Omicron, *RBD* Receptor-binding domain, S Stabilizing, *VOCs* Variants of concern

### *Ab initio* 3D modelling of the RBD region to know conformational changes

**Fig. 2:**
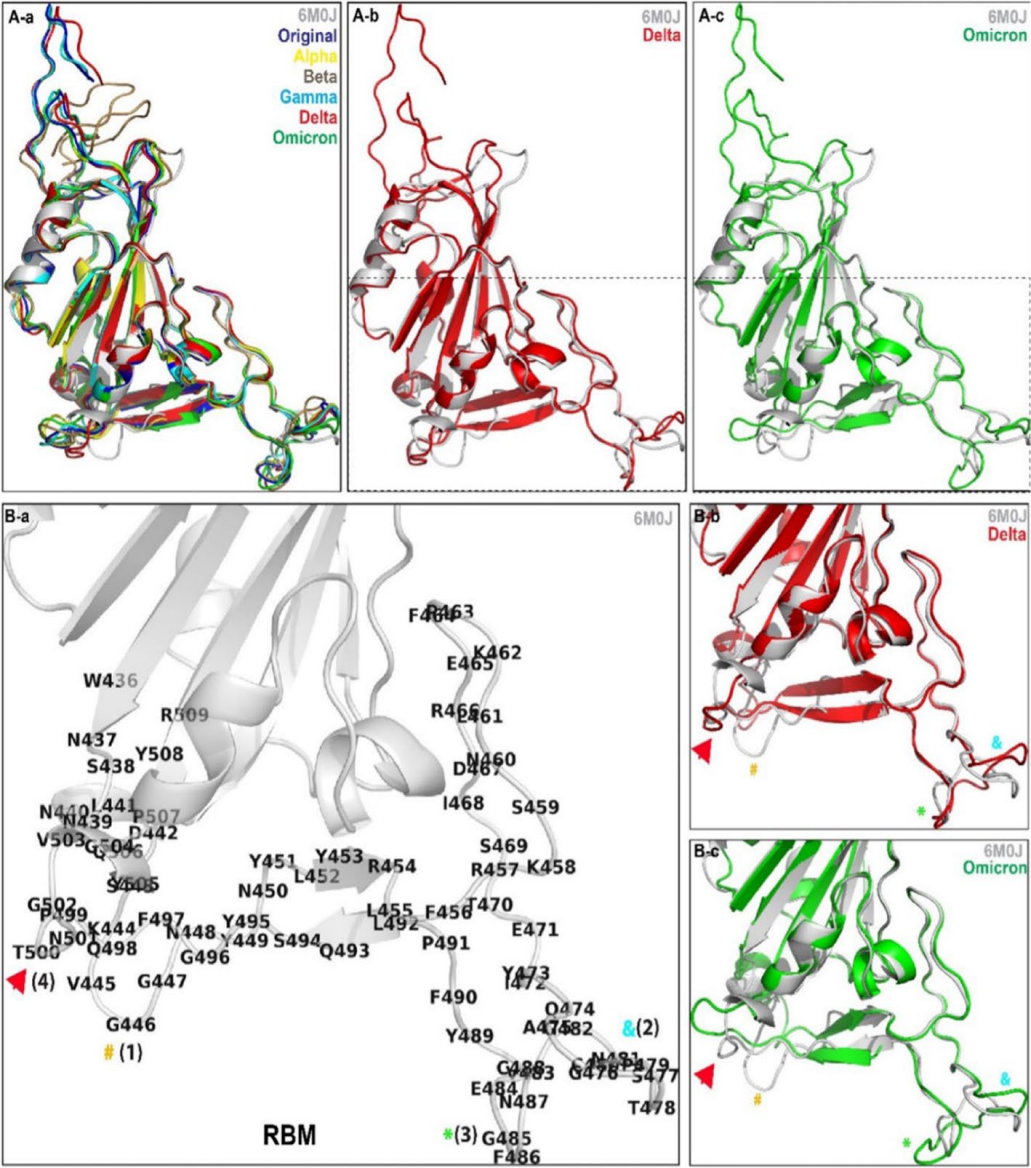
A-a The trRosetta-generated Receptor-Binding Domain (RBD) structures of all SARS CoV 2 variants were super-imposed on 6M0J. A-b Structural changes between the RBM of 6M0J and the Delta sequence. A-c Structural changes of the RBM between 6M0J and the Omicron sequence. B-a RBM of 6M0J; (1), (2), (3), (4) are the changes observed. B-b Structural changes of the RBM between 6M0J and the Delta sequence. B-c Structural changes of the RBM between 6M0J and the Omicron sequence

The primary objective of our study was to assess the trRosetta algorithm’s capability to consistently predict protein structures, particularly in scenarios where no similar structures are known or available in public databases. To achieve this, we generated predicted models for all the variants of concern using trRosetta, and these models were then superimposed onto the original Wuhan variant using the “super” command in PyMol. Upon examination, it was observed that for the majority of pairs involving trRosetta-generated models and the original Wuhan variant, the Root-Mean-Square Deviation (RMSD) ranged from 0.69 to 0.77 Å. A lower RMSD indicates a more accurate alignment of the Receptor-Binding Domains (RBDs). Consequently, we considered the trRosetta-generated models suitable for further analysis (see Table 2). Qualitative assessment of structural changes, observed through superimposing the trRosetta-generated naturally occurring variants (Fig. 2A-a), revealed that the mutations predominantly affected the interaction interface situated on the bottom section of the RBD (Fig. 2A-b, A-c). The Receptor-Binding Motif (RBM), identified as the main functional motif in the RBD, highlighted specific residues (Fig. 2B-a). Further, structural variations in comparison to Delta and Omicron sequences were noted (Fig. 2B-b, B-c), consistent with findings by Bhoumick et al., 2022 [21]. These 3D modeling outcomes unequivocally demonstrate a substantial conservation of the spike protein’s RBD. Comparing the variants to the wild type (Wuhan sequence), variations exhibit a higher alpha-helix structure, while the secondary structure prediction indicates fewer extended strands and a reduced presence of random coil shapes. The projected increase in alpha-helices suggests that beta strands are less prone to mutations than alpha helices, emphasizing the structural resilience of the spike protein’s RBD.

**Table 2:** RMSD (root mean square deviation of atomic positions) score for trRosetta-generated RBD models superimposed on original Wuhan strain. The root-mean-square deviation of atomic positions is the measure of the average distance (Å) between the atoms of superimposed proteins

Models	Root-Mean-Square Deviation (RMSD) for trRosetta-Generated Models (Å)
Original Variant	0.705 for 1434 atoms
Alpha Variant	0.694 for 1446 atoms
Beta Variant	0.777 for 1434 atoms
Gamma Variant	0.762 for 1474 atoms
Delta Variant	0.785 for 1466 atoms
Omicron Variant	0.732 for 1421 atoms

### Comparative analysis of the binding ability of mutant RBD complexes to hACE2

**Fig. 3:**
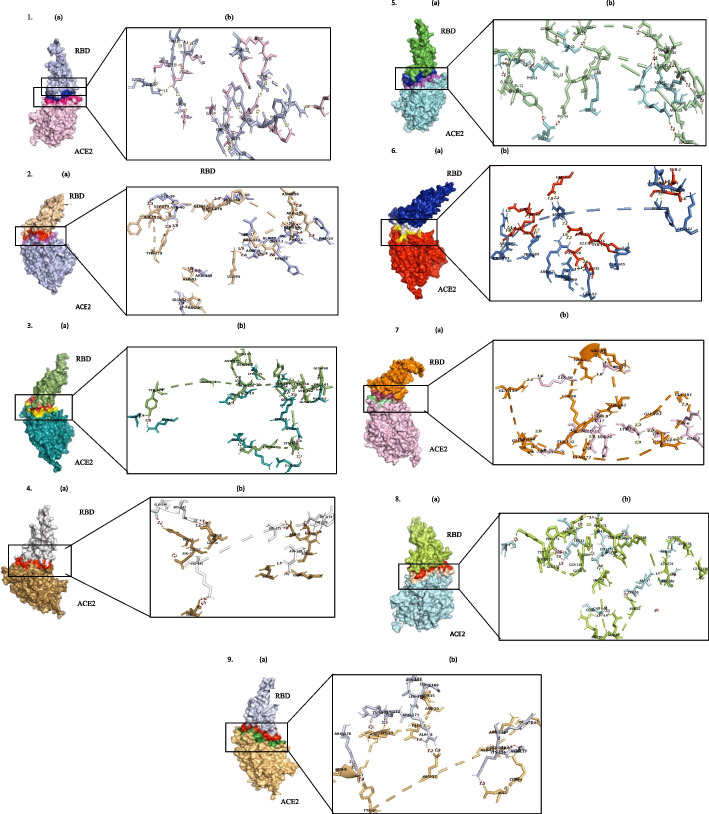
**a** Represents the binding interface of mutant complexes and a surface representation. **b** Offers the binding interface and stick model of the fundamental hydrogen bonding interactions of the mutant. (Chain A represents hACE2R, and chain B represents the RBD of each variant). Original Wuhan strain (1), Alpha variant(2), Beta variant(3), Gamma variant(4), Omicron variant BA.1.1 which has the highest binding affinity among all variants in USA (5), Omicron variant BA.1.1 which has the highest binding affinity among all variants in India (6), Delta variant B.1.617.2 which has the highest binding affinity among all variants in France (7), Omicron variant BA.1.1 which has the highest binding affinity among all variants in Germany (8), Omicron variant BA.1.1 which has the highest binding affinity among all variants in Brazil (9)

**Fig. 4:**
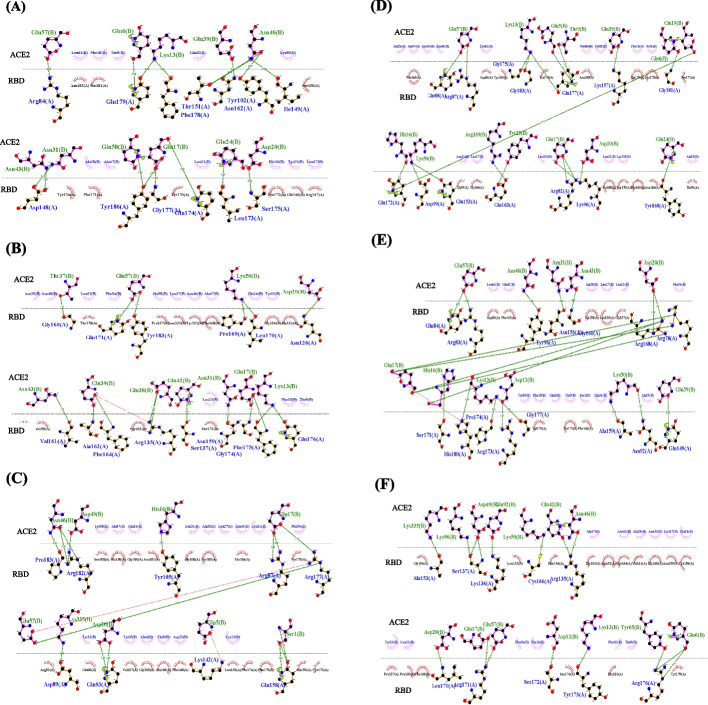
RBD's 2D DIMPOT interactions representation, including hydrogen interactions in mutant complexes. Original Wuhan strain (**A**), Omicron variant BA.1.1 which has the highest binding affinity among all variants in USA (**B**), Omicron variant BA.1.1 which has the highest binding affinity among all variants in India (**C**), Delta variant B.1.617.2 which has the highest binding affinity among all variants in France (**D**), Omicron variant BA.1.1 which has the highest binding affinity among all variants in Germany (**E**), Omicron variant BA.1.1 which has the highest binding affinity among all variants in Brazil (**F**)

The binding mechanism, affinities between Receptor-Binding Domains (RBDs) and the hACE2 receptor for various SARS-CoV-2 variants, including the original Wuhan variant, were assessed through the HADDOCK 2.0 server. The docking scores, cluster sizes, and dissociation constants (KD) were presented in the Tables 3, 4, 5, 6 and 7 for the USA, India, France, Germany, and Brazil, with corresponding docking complexes in Figs. 2D and 3 DIMPLOT representations of the interactions in Fig. 4. The original Wuhan variant’s predicted HADDOCK score was − 89.5 kcal/mol, indicating the lowest binding affinity. In the context of the five most affected countries (USA > India > France > Germany > Brazil), the Omicron BA.1.1 variant showed a remarkable 1.5-fold increase in affinity towards hACE2 in the USA (-130.1 kcal/mol), surpassing other Omicron subvariants in USA. Consistent trends emerged in binding affinity values, with the BA.1.1 variant at − 17.4 kcal/mol, indicating enhanced binding compared to the original Wuhan variant at − 12.9 kcal/mol. These findings align with data from the global COVID-19 tracker (ourworldindata.org/covid-19). Figure S4 [A, B and C]( Supplementary Data) shows COVID-19 cases and fatalities by the World Health Organization (WHO). In early 2022 Omicron outbreak (BA.1.1), USA reported the highest cases, hospitalizations, and elevated mortality, supporting the hypothesis of BA.1.1’s heightened transmissibility due to its exceptional binding affinity. In the Indian context, a comprehensive evaluation of docking scores revealed that the BA.1.1 Omicron variant displayed a 1.5-fold increase in binding affinity at -114 kcal/mol towards the human ACE2 receptor (hACE2). This heightened affinity closely mirrored that of the Delta variant, at -112.4 +/- 3.7 kcal/mol. Importantly, the computed docking scores aligned with the corresponding binding affinity with the BA.1.1 variant showing an affinity of -15.2 kcal/mol, not significantly different from the Delta variant B.1.617.2’s -14.8 kcal/mol. Additionally, an analysis of global COVID-19 tracker data reinforced these findings. Figure S5 [A, B and C] ( Supplementary Data) depicted the extensive incidence of confirmed COVID-19 cases and fatalities, sourced from World Health Organization (WHO). During mid-2021 Delta variant B.1.617.2 outbreak, India witnessed a devastating initial COVID-19 wave marked by significant fatalities, a surge in cases, hospitalizations, and elevated mortality. Remarkably, vaccines were not widely accessible during this period, emphasizing the pivotal role of the Delta variant’s exceptional binding affinity in driving its heightened transmissibility and virulence, culminating in a significant impact of SARS-CoV-2 in India. In contrast, in the French context, the Delta variant B.1.617.2 had the highest HADDOCK score, registering at -102.3 +/- 21.4 kcal/mol, closely resembling the Gamma variant P.1’s HADDOCK score at -98.1 +/- 9.5 kcal/mol. These scores directly correlated with binding affinity values of -14.4 kcal/mol and − 14.3 kcal/mol. Furthermore, analysis of global COVID-19 tracker data supported these findings. Figure S6 [A, B and C] ( Supplementary Data) graphically displayed the extensive confirmed COVID-19 cases and fatalities during the late 2021 and early 2022 Delta variant B.1.617.2 outbreak in France, offering compelling evidence for the Delta variant’s substantial transmissibility and virulence during this period in the country. Similar findings were observed in both Germany and Brazil, mirroring those seen in the USA. The BA.1.1 Omicron variant exhibited a 1-fold increase in binding affinity compared to the original Wuhan strain with HADDOCK scores ranging from − 96.7 +/- 4.6 kcal/mol to -116.5 +/- 2.0 kcal/mol and binding affinity from − 16.5 kcal/mol and − 15.4 kcal/mol, respectively. Global COVID-19 tracker data aligns with our study’s findings, as depicted in Figure S7 [A, B and C] ( Supplementary Data) and Figure S8 [A, B and C] ( Supplementary Data). This representation illustrates the comprehensive landscape of confirmed COVID-19 cases and confirmed deaths, sourced from WHO records. During the early 2022 Omicron variant BA.1.1 outbreak, both Germany and Brazil experienced significant COVID-19 outbreaks, characterized by a substantial loss of lives, increased confirmed cases, hospitalizations, and elevated mortality rates. This underscores the notion that the exceptional binding affinity of the Omicron variant significantly contributed to its peak transmissibility and virulence during this period in both Germany and Brazil. The ongoing evolution of SARS-CoV‐2 has resulted in considerable genetic diversity within the viral population. The virus gains entry into host cells by interacting with the hACE‐2 receptor, a process facilitated by the binding of the Receptor-Binding Domain (RBD) of the spike protein (SP). Mutations in the spike protein have the potential to enhance viral entry, and alterations in the RBD can impact viral infectivity and stability [22]. Consequently, the interaction interface between the spike protein and the hACE‐2 receptor has become a key focus in the development of novel drugs [23]. This study aims to investigate how mutations in the RBD influence binding affinity and stability across various Variants of Concern (VOCs), ranging from the wild type (WT) to the Omicron variant. Notably, variants originating in the United Kingdom (Alpha‐ B.1.1.7), South Africa (Beta‐ B.1.351), and Brazil exhibit significant structural differences and altered binding properties [24]. As an example, the study identified a specific mutation (N501Y) in the RBD of the Alpha variant compared to the wild type (WT). This mutation represents one of several alterations that researchers are exploring to understand how changes in the RBD may impact the virus’s ability to bind to and enter host cells. The research seeks to provide insights into the dynamic relationship between RBD mutations and their effects on viral infectivity, shedding light on potential targets for drug development and therapeutic interventions. The alteration introduced in the Alpha variant increased its binding capacity by 7.9% compared to the wild type (WT) [25], potentially contributing to the higher reported cases of COVID-19 associated with the Alpha variant. Additionally, we noted variations in salt bridges and hydrogen bonds among different variants, such as the absence of a salt bridge involving Lys478 in Alpha, Beta, and Gamma complexes but its presence in the BA.5 variant. This suggests a potential link between these mutations and variant transmissibility [26]. In our study, we observed the formation of a salt bridge between K417 (RBD) and D38 (hACE‐2) in the WT RBD-hACE2 complex, confirming the results obtained by Wang et al. [27]. However, the substitution of K417 by N417 and T417 in Beta and Gamma, respectively, resulted in the loss of the salt bridge. In contrast to observations by Han et al. [28] and Li et al. [29], where the absence of salt bridges resulted in reduced binding affinity for hACE‐2, our results contradicted their findings. We observed a high binding energy for the Gamma variant compared to the wild type (WT), even in the absence of salt bridge formation. This underscores the significance of other intermolecular interactions in enhancing binding capacities. The binding affinity (∆G‐kcal/mol) plays a crucial role in determining whether complex formation occurs, holding a vital role in controlling interactions. With the exception of Delta and Omicron, other Variants of Concern (VOCs) displayed higher KD and weaker binding affinity values, aligning with the results reported by Khan et al. [24]. In our study, BA.2.12.1 exhibited the lowest binding affinity (-13.6 kcal/mol), indicating high stability of the complex compared to other variants. BA.2.12.1 also demonstrated the lowest KD value (9.8 × 10 − 11) and high hACE‐2 affinity compared to other VOCs and Omicron subvariants. A reduced KD value signifies a strong binding between the spike protein-RBD and hACE‐2, establishing a greater affinity between the receptor and ligand [30]. It is noteworthy that among Alpha, Beta, Gamma, and Omicron variants, the N501Y mutation in their RBDs is a primary concern, as it is one of the active residues directly interacting with hACE2R.The HADDOCK scores highlight that both the Delta variant (-99 kcal/mol) and the Omicron subvariants (OSVs) exhibit more robust binding abilities compared to the wild type (WT) (-87 kcal/mol). The Receptor Binding Domain (RBD) of Omicron (B.1.1.529) has undergone notable mutations. Particularly, the BA.1 and BA.2 OSVs, aligning with findings by Han et al. [31], are more prevalent and display the lowest score (-130.1 kcal/mol) in the case of BA.2. This data reinforces the higher transmissibility observed in the Omicron variant and its subvariants [32]. Studies indicate that the BA.2 Omicron subvariant is 1.5 times more contagious than BA.1, substantiating its high binding energy [33]. Additionally, Omicron BA.1 is predicted to be significantly more contagious than Wuhan‐Hu‐1 and Delta variants, primarily attributed to RBD mutations N440K, T478K, and N501Y [34]. Omicron demonstrates an overall infectivity level that surpasses the ancestral SARS‐CoV‐2 variation and other subsequent variants, including Delta, mainly attributed to its extensive mutations in the Receptor Binding Domain (RBD) region [35]. The docking scores for Delta and Alpha exhibit similarities to those of BA.5, potentially due to amino acid substitutions at L452R and T478K. Notably, these mutations, along with F486V, are absent in BA.2. Similar to Delta, the 478th residue in the RBD of BA.5 forms a salt bridge and hydrogen bond with the human angiotensin‐converting enzyme receptor (hACE2R). In the context of the Delta variant, the presence of the Arg452 mutation is also observed in BA.5. In BA.5, this mutation contributes to the formation of two hydrogen bonds and one salt bridge, leading to increased binding affinity compared to Delta. Notably, despite BA.5 having lower binding affinity than BA.2, it demonstrates heightened pathogenicity and efficient transmission in humans, suggesting that factors beyond binding energy play a role in viral transmission. When examining the active site residues of the Receptor Binding Domains (RBDs), residue 493 displayed hydrogen bond formations in Other SARS-CoV-2 variants (OSVs) complexes, except in BA.2 and BA.5. The formation of hydrogen bonds is crucial for the docking score value, highlighting their significance in the overall interaction dynamics. Hence the binding stability of hACE2 and RBD complexes were in the order of Omicron BA.1.1 > Omicron BA.5.2.1 > Omicron BA.2.12.1 > Delta > Omicron-B.1.529.1 > Gamma > Alpha > Beta > Original Wuhan strain due to the increase of the KD.

**Table 3:** HADDOCK predicted docking results between human ACE2 and RBD complexes of variant of concern and original Wuhan strain in USA

Protein -protein complex	Date of sample collected	HADDOCK Score (Kcal/mol)	Cluste Size	Z-Score	Binding affinity ΔG (kcal mol-1)	Dissociation Constant Kd (M) at ℃
**Wuhan**	01-12-2019	-89.5 +/- 1.8	86	-1.5	-12.9	3.7E-10
**A-P518T**	30-01-2021	-112.6 +/- 12.3	42	-2.1	-15.1	7.7E-12
**A-Y498N**	02-02-2021	-64.8 +/- 5.6	36	-1.2	-13	2.8E-10
**A-G444V**	08-02-2021	-108.8 +/- 6.1	54	-2	-13	3E-10
**A- F426S**	24-02-2021	-97.4 +/- 9.5	24	-2.1	-14.3	3.3E-11
**A-E484K**	02-03-2021	-100.1 +/- 5.8	37	-2.6	-13.9	6.6E-11
**A-R400S**	10-03-2021	-88.9 +/- 2.4	59	-1	-12.1	1.4E-09
**A-R343T**	18-03-2021	-88.0 +/- 7.1	12	-1.5	-13.1	2.7E-10
**A-S491P**	22-04-2021	-73.9 +/- 5.0	32	-2.1	-14	5.2E-11
**A-L438R**	28-04-2021	-89.5 +/- 3.3	33	-2.5	-13.5	1.3E-10
**A-N437K**	17-06-2021	-73.7 +/- 4.5	27	-1.5	-13.1	2.5E-10
**A-H516Y**	28-06-2021	-67.4 +/- 4.9	19	-1.6	-10.9	0.000000011
**A-Q490R**	20-07-2021	-84.0 +/- 7.4	63	-1.4	-12.9	3.3E-10
**A-F487S**	20-07-2021	-93.8 +/- 3.2	163	-1.4	-13.1	2.7E-10
**A-A517S**	02-07-2021	-81.3 +/- 5.0	34	-1.9	-13.7	8.3E-11
**A-E403Q**	04-08-2021	-83.4 +/- 7.3	24	-1.8	-13.2	2.1E-10
**A-K441M**	09-09-2021	-99.1 +/- 0.7	152	-1.7	-13.7	8.7E-11
**A-Y486H**	09-09-2021	-101.0 +/- 8.5	13	-1.4	-13	2.8E-10
**B-E484K** **B-N501Y**	09-10-2020	-96.6 +/- 6.8	46	-2.3	-12	1.6E-09
**B-N501K**	13-05-2021	-85.0 +/- 11.0	16	-1.5	-13.1	2.3E-10
**B-G339V**	09-10-2020	-96.4 +/- 12.5	4	-1.2	-16.9	4E-13
**G- E484K** **G- N501Y**	15-03-2020	-72.8 +/- 3.0	90	-1.5	-10.6	0.000000016
**G-K417T**	06-07-2021	-64.4 +/- 9.7	25	-1.9	-12.5	6.6E-10
**G-D427N**	27-04-2021	-63.4 +/- 1.9	100	-0.7	-11.8	2.2E-09
**G-T323I**	28-04-2021	-90.5 +/- 3.9	51	-1.5	-11.3	5.5E-09
**D-L452R**	17-05-2021	-95.0 +/- 3.0	73	-1.6	-13.7	8.6E-11
**D-T478K** **D-N501Y**	17-05-2021	-71.0 +/- 18.7	4	-1.3	-12.6	6.2E-10
**D-E324Q**	16-07-2021	-90.9 +/- 4.3	44	-1.7	-11.2	5.9E-09
**D-T323I**	04-08-2021	-51.0 +/- 8.5	42	-1.4	-8.9	0.00000028
**D-G446V**	14-08-2021	-90.9 +/- 2.3	14	-1.5	-13.3	1.7E-10
**D-A520S**	28-10-2021	-58.4 +/- 17.1	4	-1.7	-13.5	1.2E-10
**O1-G342D**	25-12-2021	-74.4 +/- 3.9	49	-1.7	-8.4	0.00000071
**O1-Q496R**	04-01-2022	-79.3 +/- 9.4	6	-0.6	-11.1	6.8E-09
**O1-G449S**	04-01-2022	-102.2 +/- 2.2	176	-1	-13.4	1.4E-10
**O1-D408N**	30-01-2022	-111.6 +/- 3.2	18	-1.4	-15.4	5.4E-12
**O1-S480N**	19-04-2022	-110.5 +/- 4.3	63	-1.5	-13	3E-10
**O1-S374F**	19-07-2022	-105.4 +/- 5.4	38	-1.3	-12.6	5.8E-10
**O1-R349T**	10-11-2022	-90.6 +/- 4.9	10	-1.8	-16	1.7E-12
**O1-T526I**	16-08-2022	-79.5 +/- 8.8	4	-2	-11.1	7.2E-09
**O2-N415K**	02-01-2021	-110.6 +/- 0.8	60	-1.5	-14.6	2E-11
**O2-K438N**	12-01-2022	-96.9 +/- 3.9	60	-0.8	-12.9	3.3E-10
**O2-N458K**	28-02-2022	-92.3 +/- 3.5	43	-1	-12.4	7.5E-10
**O2-T428I**	04-03-2022	-110.6 +/- 10.2	5	-1.7	-15.2	7.5E-12
**O2-N352K**	19-03-2022	-81.8 +/- 2.6	14	-1.2	-14.2	3.7E-11
**O2-R491Q**	**16-09-2022**	**−130.1+/- 8.1**	**19**	**-1**	**-17.4**	**1.7E-13**
**O2-K476T**	27-10-2022	-93.2 +/- 5.3	92	-1.3	-13	2.9E-10
**O2-F484P**	10-06-2022	-88.6 +/- 14.3	5	-1.4	-13.8	7.5E-11
**O2-K344E**	22-07-2022	-119.7 +/- 9.6	92	-1.7	-14.8	1.3E-11
**O2-D337N**	30-07-2022	-51.3 +/- 1.8	22	-2	-9.1	0.00000023
**O2-Y447S**	18-07-2022	-113.7 +/- 6.6	14	-1.3	-14.8	1.3E-11
**O2-L453S**	21-08-2022	-94.8 +/- 2.9	124	-1.4	-13.4	1.6E-10
**O2-N475S**	20-07-2022	-51.4 +/- 5.9	7	-1.7	-13	2.9E-10
**O2-K476E**	20-12-2022	-108.4 +/- 2.3	92	-1.5	-13.1	2.3E-10
**O2-N475D**	01-12-2022	-94.4 +/- 5.1	24	-1.2	-13	2.9E-10
**O3-H501Y**	28-09-2021	-108.0 +/- 22.8	4	-1	-14.5	2.5E-11
**O3-A376T**	23-12-2021	-88.5 +/- 9.9	31	-1.4	-13.1	2.5E-10
**O3-D339G**	07-02-2022	-78.6 +/- 7.9	32	-1.3	-10.2	0.00000003
**O3-R346T**	25-05-2022	-86.3 +/- 8.8	19	-2.4	-12.6	5.7E-10
**O3-D339Y**	31-08-2022	-107.9 +/- 5.6	107	-1.7	-15.9	2.2E-12
**O3-D339H**	21-09-2022	-67.5 +/- 5.5	66	-1.9	-12.7	4.5E-10
**O3-A484G**	30-09-2022	-93.0 +/- 2.4	31	-1.7	-13.6	1.1E-10
**O4-S405R**	04-05-2022	-111.1 +/- 4.9	95	-1.3	-14.8	1.5E-11
**O4-Q488R**	15-05-2022	-106.1 +/- 8.4	35	-1.4	-15.2	7.7E-12
**O4-N400D**	19-05-2022	-96.1 +/- 7.1	31	-1.3	-13.5	1.2E-10
**O4-T467I**	03-09-2022	-95.8 +/- 5.7	35	-1.9	-13.2	2.2E-10
**O4-K441T**	01-11-2022	-65.1 +/- 0.3	27	-1.1	-12.5	6.7E-10
**O4-R343T**	20-12-2022	-124.8 +/- 9.0	31	-1.9	-12.5	7.4E-10
**O4-K437N**	15-12-2022	-74.5 +/- 11.8	7	-1.3	-13.1	2.4E-10
**O4-N457K**	23-12-2022	-64.4 +/- 2.5	30	-1.3	-11.4	4.7E-09
**O4-N447D**	26-12-2022	-80.2 +/- 6.3	24	-1.9	-13.1	2.4E-10
**O4-D417N**	03-01-2023	-102.3 +/- 2.0	52	-2	-12.7	5.2E-10
**O4-N412K**	13-01-2023	-106.4 +/- 9.4	9	-1.4	-15.8	2.7E-12
**O4-A432S**	18-01-2023	-88.3 +/- 5.6	73	-1.9	-14.1	4.3E-11
**O4-D336H**	27-01-2023	-119.3 +/- 9.7	11	-1.5	-15.8	2.7E-12

**Table 4:** HADDOCK predicted docking results between human ACE2 and RBD complexes of Variants of concern and original Wuhan strain in India

Protein -protein complex	Date of sample collected	HADDOCK Score (Kcal/mol)	Cluster size	Z-Score	Binding affinity ΔG (kcal mol-1)	Dissociation Constant Kd (M) at ℃
**I-A-N501Y**	2020-11-27	-112.7 +/- 7.9	10	-2.4	-13.8	6.9E-11
**I-A-E484K**	2021-04-07	-104.1 +/- 13.0	11	-1.9	-13.8	6.9E-11
**I-B-K417N**	2021-02-02	-106.8 +/- 8.9	48	-1.7	-15.1	2.6E-12
**I-D-E484Q**	2021-05-22	-74.9 +/- 8.6	97	-1.5	-10.4	0.000000023
**I-D-L452R**	2021-03-21	-112.4 +/- 3.7	149	-1.4	-14.6	1.8E-11
**I-D-G446D**	2021-07-21	-98.0 +/- 5.6	82	-2	-10.6	0.000000018
**I-D-E471Q**	2021-07-14	-87.4 +/- 2.9	11	-1.6	-10	0.000000047
**I-D-L512H**	2021-07-15	-76.7 +/- 2.2	75	-2.5	-14.8	1.5E-11
**I-D-E516Q**	2021-07-28	-83.5 +/- 5.6	29	-1.6	-13.2	2E-10
**I-D-I410S**	2021-09-01	-82.0 +/- 3.7	102	-1.4	-11.1	7.3E-09
**I-D-N360Y**	2021-09-01	-94.0 +/- 3.3	75	-1.8	-14	5.1E-11
**I-O1-G342D**	**2021-11-20**	**-114.7**	**5**	**-1.5**	**-15.2**	**7.3E-12**
**I-D-S477G**	2021-09-10	-90.1 +/- 6.3	45	-1.7	-10.6	0.000000017
**I-D-E484K**	2021-09-25	-79.0 +/- 6.7	28	-1.3	-14	5.6E-11
**I-D-S494L**	2021-10-04	-103.9 +/- 3.1	15	-1.8	-12	1.6E-09
**I-D-N501Y**	2021-10-04	-93.6 +/- 7.0	13	-1.6	-13.4	1.5E-10

**Table 5:** HADDOCK predicted docking results between human ACE2 and RBD complexes of variants of concern and original Wuhan strain in France

Protein -protein complex	Date of sample collected	HADDOCK Score (Kcal/mol)	Cluster size	Z-Score	Binding affinity ΔG (kcal mol-1)	Dissociation Constant Kd (M) at ℃
**F-A-Y449S**	2021-06-30	-96.0 +/- 2.6	31	-1.4	-13.5	1.3E-10
**F-A-A522S**	2021-02-02	-90.4 +/- 5.4	13	-2	-13.8	7.3E-11
**F-B-K417N**	2021-01-18	-75.6 +/- 10.9	11	-1.9	-13.6	1.1E-10
**F-G-K417T**	2021-01-27	-98.1 +/- 9.5	42	-2.1	-14.3	1.6E-11
**F-D-L452R**	2021-06-09	-102.3 +/- 21.4	76	-1.6	-14.4	2.6E-11
**F-D-A411S**	2021-07-05	-72.5 +/- 6.1	20	-2.2	-11.3	5.4E-09
**F-D-N360K**	2021-12-06	-102.8 +/- 1.0	60	-2.1	-14.1	4.7E-11
**F_A_A520S**	2021-05-18	-102.7 +/- 5.6	52	-1.2	-13.7	8.3E-11
**F_A_F490S**	2021-07-25	-71.0 +/- 14.9	10	-1.6	-14.1	1.6E-11
**F-O1-G342D**	**2021-11-20**	**-114.7**	**5**	**-1.5**	**-15.2**	**7.3E-12**
**F-D-E484Q**	2021-05-22	-79.6 +/- 1.8	49	-1.2	-10.4	0.000000023

**Table 6:** HADDOCK predicted docking results between human ACE2 and RBD complexes of variants of concern and original Wuhan strain in Germany

Protein -protein complex	Date of sample collected	HADDOCK Score (Kcal/mol)	Cluster size	Z-Score	Binding affinity ΔG (kcal mol-1)	Dissociation Constant Kd (M) at ℃
G-O-G339D	2022	-109.2 +/- 7.7	8	-1.3	-15.5	4.1E-12
**G-O-S371F**	2022-10-05	-96.7 +/- 4.6	5	-1.2	-15.4	5.3E-12
**G_A_N501Y**	2020-11-27	-112.7 +/- 7.9	10	-2.4	-13.8	6.9E-11
**F_B_K417N**	2021-01-12	-106.8 +/- 8.9	48	-1.7	-13.6	1.1E-10
**F_G_K417T**	2021-02-25	-98.1 +/- 9.5	42	-2.1	-14.7	1.6E-11
**F_D_L452R**	2021-04-14	-112.4 +/- 3.7	149	-1.4	-14.4	2.6E-11

**Table 7:** HADDOCK predicted docking results between ACE2 and RBD complexes of variants of concern and original Wuhan strain in Brazil

Protein -protein complex	Date of sample collected	HADDOCK Score (Kcal/mol)	Cluster size	Z-Score	Binding affinity ΔG (kcal mol-1)	Dissociation Constant Kd (M) at ℃
**B_A_N501Y**	2020-11-27	-112.7 +/- 7.9	10	-2.4	-13.8	6.9E-11
**B-G-A348S**	2021-03-06	-73.0 +/- 4.6	170	-1.6	-11.6	0.000000003
**B-G-A344S**	2021-05-11	-64.7 +/- 6.1	66	-1.5	-10.6	0.000000017
**B-G-D427N**	2021-07-07	-105.2 +/- 2.7	191	-1	-13.7	9.2E-11
**B-G-P330S**	2021-09-02	-77.4 +/- 6.6	12	-1.6	-13.9	6.1E-11
**B-G-S373L**	2021-09-09	-73.5 +/- 3.4	11	-1.5	-14.5	2.3E-11
**B-G-G413W**	2021-08-11	-108.7 +/- 8.0	15	-2.2	-14.5	2.3E-11
**B_G_K417T**	2020-04-29	-98.1 +/- 9.5	42	-2.1	-14.7	1.6E-11
**B_G_T323I**	2021-07-12	-90.5 +/- 3.9	51	-1.5	-11.3	5.5E-09
**B_D_L452R**	2021-07-01	-112.4 +/- 3.7	149	-1.4	-14.4	2.6E-11
**B_D_E452Q**	2021-05-22	-79.6 +/- 1.8	49	-1.2	-10.4	0.000000023
**B_D_A520S**	2021-12-07	-58.4 +/- 17.1	4	-1.7	-13.5	1.2E-10
**B-O-G339D**	**2021-12-12**	**-116.5 +/- 2.0**	**168**	**-1.3**	**-16.7**	**5.6E-13**
**G_O3_S317F**	2022-10-05	-96.7 +/- 4.6	5	-1.2	-15.4	5.3E-12

## Conclusion

We examined five variants of concern (VOCs), in addition to the wild type (WT), and four subvariants of Omicron (OSVs) to evaluate changes in conformation and their impact on protein stability. The heightened quantity and quality of interactions between the spike protein (SP)-RBDs of Omicron and hACE2R suggest increased potency, providing a biophysical rationale for the heightened transmissibility of OSVs. Our results indicate that mutations in active RBD residues lead to enhanced binding affinity and intermolecular interactions in mutant complexes, supporting the elevated transmissibility of the Omicron variant. Furthermore, through the analysis of intermolecular interactions in diverse variant complexes, this study may establish a solid foundation for structure-based drug design targeting OSVs.

### Supplementary Information

**Supplementary Material 1.**

## Data Availability

Data is provided within the manuscript or supplementary information files.
