# Supplementary material for: Computational analysis of affinity dynamics between the variants of SARS-CoV-2 spike protein (RBD) and human ACE-2 receptor
Source: Virol J. 2024 Apr 19;21:88. doi: 10.1186/s12985-024-02365-3 (PMC11031966; doi:10.1186/s12985-024-02365-3)
Supplement: Supplementary file 1 — Supplementary Material 1. [file 12985_2024_2365_MOESM1_ESM.docx]

**Supplementary material**

**Table S1: Mutations noted in the Alpha variant (B.1.1.7).**

| **SI no.** | **Country** | **Variant** | **Total no. of sequences** | **Total no. of Mutations** | **Mutations** |
| --- | --- | --- | --- | --- | --- |
| 1 | USA | Alpha (B.1.1.7) | 1471 | 17 | P518T, Y498N, G444V, F426S, E484K, R400S, R343T, S491P, L438R, N437K, H516Y, Q490R, F487S, A517S, E403Q, K441M, Y486H |
| 2 | India | Alpha (B.1.1.7) | 60 | 2 | N501Y, E484K |
| 3 | France | Alpha (B.1.1.7) | 277 | 6 | N501Y, A522S, A520S, E484K, Y449S, F490S |
| 4 | Germany | Alpha (B.1.1.7) | 4 | 1 | N501Y |
| 5 | Brazil | Alpha (B.1.1.7) | 29 | 1 | N501Y |

**Table S2: Mutations noted in the Beta variant (B.1.351).**

| **SI no.** | **Country** | **Variant** | **Total no. of sequences** | **No. of mutations** | **Mutations** |
| --- | --- | --- | --- | --- | --- |
| 1 | USA | Beta (B.1.351) | 473 | 4 | K417N, E484K, N501Y, G339V |
| 2 | India | Beta (B.1.351) | 9 | 3 | K417N, E484K, N501Y |
| 3 | France | Beta (B.1.351) | 41 | 3 | K417N, E484K, N501Y |
| 4 | Germany | Beta (B.1.351) | 7 | 3 | K417N, E484K, N501Y |
| 5 | Brazil | Beta (B.1.351) | - | - | - |

**Table S3: Mutations noted in the Gamma variant (P.1).**

| **SI no.** | **Country** | **Variant** | **Total no. of sequences** | **No. of mutations** | **Mutations** |
| --- | --- | --- | --- | --- | --- |
| 1 | USA | Gamma (P.1) | 238 | 5 | K417N, E484K, N501Y, D427N, T323I |
| 2 | India | Gamma (P.1) | - | - | - |
| 3 | France | Gamma (P.1) | 18 | 3 | K417T, E484K, N501Y |
| 4 | Germany | Gamma (P.1) | 5 | 3 | K417T, E484K, N501Y |
| 5 | Brazil | Gamma (P.1) | 225 | 11 | K417T, E484K, N501Y, A348S, A344S, A520S, D427N, T323I, P330S, S373L, G413W |

**Table S4: Mutations noted in the Delta variant (B.1.617.2).**

| **SI no.** | **Country** | **Variant** | **Total no. of sequences** | **No. of mutations** | **Mutations** |
| --- | --- | --- | --- | --- | --- |
| 1 | USA | Delta  (B.1.617.2) | 401 | 9 | L452R, T478K, E484Q, N501Y, E324Q, T323I, G446V, A520S, F490S |
| 2 | India | Delta  (B.1.617.2) | 265 | 13 | L452R, T478K, E484Q, G446D, E471Q, L517H, E516Q, I410S, N460Y, S477G, S359G, S494L, N501Y |
| 3 | France | Delta  (B.1.617.2) | 255 | 8 | L452R, T478K, E484Q, A411S, S494L, T323I, A520S, N360K |
| 4 | Germany | Delta  (B.1.617.2) | 44 | 2 | L452R, T478K |
| 5 | Brazil | Delta  (B.1.617.2) | 15 | 4 | L452R, T478K, E484Q, A411S |

**Table S5: Mutations noted in the Omicron and its sub variants.**

| **SI no.** | **Country** | **Variant** | **Total no. of sequences** | **No. of mutations** | **Mutations** |
| --- | --- | --- | --- | --- | --- |
| 1 | USA | Omicron  B.1.1.529  BA.1.1  BA.2.12.1  BA.5.2.1  B.1.617.2 |  | 23  23  17  18  13 | G339D, S371L, S373P, S375F, K417N, N440K, G446S, T478K, E484A, Q493R, G496S, N501Y, Y505H, D405N, R408S, S478N, L452Q, Q498R, S371F, T376A, R346T, L452R, T523I  N415K, K438N, S444G, N45K, T428I, N352K, R491Q, S494G, Y499N, H503Y, R496Q, K476T, A482E, F488S, F484P, K344E, D337N, K442S, Y447S, L453S, N475S, K476E, N475D  H505Y, A376T, K440N, D339G, F371S, P373S, F375S, N405D, S408R, N417K, L452Q, K440N, R346T, D339Y, R493Q, D339H, A482G  S86R, N93K, Q169R, N81D, K116N, R128L, T148I, K122T, G124R, R24T, K118N, R130L, N138K, N128D, D98N, N95K, A113, SD17H  L452R, T478K, N501Y, T323I, G446V, K417N, R355S, D427A, A520S, E484K, S373P, F490S, E484A |
| 2 | India | Omicron (BA.1.1)  B.1.617.2 | 26  260 | 14  11 | G339D, S371L, S373P, S375F, K417N, N440K, G446S, T478K, E484A, Q498R, G496S, Q498R, N501Y, Y505H  G446D, E471Q, L517H, E516Q, I410S, N460Y, S477G, S359G, E484K, S494L, N501Y |
| 3 | France | Omicron (BA.1.1)  B.1.617.2 | 56  243 | 15  7 | G339D, S371L, S373P, S375F, K417N, N440K, G446S, T478K, E484A, Q498R, G496S, Q498R, N501Y, Y505H  L452R, T478K, A411S, T323I, N360K, E484Q, A520S |
| 4 | Germany | Omicron (BA.1.1)  B.1.617.2  BA.5.2.1 | 6  44  3 | 15  6  10 | G339D, S371L, S373P, S375F, K417N, N440K, G446S, T478K, E484A, Q498R, G496S, Q498R, N501Y, Y505H  L452R  G339D, S371F, S373P, S375F, T376A,  D405N, R408S, K417N, N440K, L452R, T478K, E484A, Q498R, N501Y, Y505H |
| 5 | Brazil | Omicron  (BA.1.1)  B.1.617.2  BA.5.2.1 | 36  15  9 | 14  4  14 | G339D, S371L, S373P, S375F, K417N, N440K, G446S, T478K, E484A, Q498R, G496S, Q498R, N501Y, Y505H  L452R, T478K, E484Q, A520S  G339D, S371L, S373P, S375F, K417N, N440K, G446S, T478K, E484A, Q498R, G496S, Q179R, N182Y, Y186H |

**Table S6: List of amino acid sequences of S protein of SAR COV-2 variants retrieved from NCBI along with the country isolated and pango lineage.**

| **SI No.** | **Country** | **Variant** | **Time Period** | **No. of sequences collected** |
| --- | --- | --- | --- | --- |
| 1 | China | Wuhan | 1^st^ Dec. 2019 – 28^th^ Feb. 2023 | 11 |
| 2 | USA | Alpha  (B.1.1.7) | 1/12/2019 – 28/2/2020  1/3/2020- 30/5/2020  1/6/2020- 30/8/2020  1/9/2020-30/11/2020  1/12/2020-28/2/2021  1/3/2021-30/5/2021  1/6/2021-30/8/2021  1/9/2021-30/11/2021  1/12/2021-28/2/2022  1/3/2022-30/5/2022  1/6/2022-30/8/2022  1/9/2022-30/11/2022  1/12/2022-28/2/2023 | 0  1  0  1  500  500  500  36  16  0  0  0  0 |
|  | USA | Beta  (B.1.351) | 1/12/2019 – 28/2/2020  1/3/2020- 30/5/2020  1/6/2020- 30/8/2020  1/9/2020-30/11/2020  1/12/2020-28/2/2021  1/3/2021-30/5/2021  1/6/2021-30/8/2021  1/9/2021-30/11/2021  1/12/2021-28/2/2022  1/3/2022-30/5/2022  1/6/2022-30/8/2022  1/9/2022-30/11/2022  1/12/2022-28/2/2023 | 0  0  0  1  43  341  23  0  0  0  0  0  0 |
|  | USA | Gamma  (P.1) | 1/12/2019 – 28/2/2020  1/3/2020- 30/5/2020  1/6/2020- 30/8/2020  1/9/2020-30/11/2020  1/12/2020-28/2/2021  1/3/2021-30/5/2021  1/6/2021-30/8/2021  1/9/2021-30/11/2021  1/12/2021-28/2/2022  1/3/2022-30/5/2022  1/6/2022-30/8/2022  1/9/2022-30/11/2022  1/12/2022-28/2/2023 | 0  1  0  1  21  100  100  19  0  0  0  0  0 |
|  | USA | Delta  (B.1.617.2) | 1/12/2019 – 28/2/2020  1/3/2020- 30/5/2020  1/6/2020- 30/8/2020  1/9/2020-30/11/2020  1/12/2020-28/2/2021  1/3/2021-30/5/2021  1/6/2021-30/8/2021  1/9/2021-30/11/2021  1/12/2021-28/2/2022 1/3/2022-30/5/2022  1/6/2022-30/8/2022  1/9/2022-30/11/2022  1/12/2022-28/2/2023 | 0  0  0  0  0  100  100  100  100  0  0  0  0 |
|  | USA | Omicron  (B.1.1.529) | 1/12/2019 – 28/2/2020  1/3/2020- 30/5/2020  1/6/2020- 30/8/2020  1/9/2020-30/11/2020  1/12/2020-28/2/2021  1/3/2021-30/5/2021  1/6/2021-30/8/2021  1/9/2021-30/11/2021  1/12/2021-28/2/2022  1/3/2022-30/5/2022  1/6/2022-30/8/2022  1/9/2022-30/11/2022  1/12/2022-28/2/2023 | 0  0  0  1  0  0  0  0  0  100  100  31  5 |
|  | USA | Omicron  (BA.1.1) | 1/12/2019 – 28/2/2020  1/3/2020- 30/5/2020  1/6/2020- 30/8/2020  1/9/2020-30/11/2020  1/12/2020-28/2/2021  1/3/2021-30/5/2021  1/6/2021-30/8/2021  1/9/2021-30/11/2021  1/12/2021-28/2/2022  1/3/2022-30/5/2022  1/6/2022-30/8/2022  1/9/2022-30/11/2022  1/12/2022-28/2/2023 | 0  0  0  0  9  1  3  13  100  100  50  9  8 |
|  | USA | Omicron  (BA.2.12.1) | 1/12/2019 – 28/2/2020  1/3/2020- 30/5/2020  1/6/2020- 30/8/2020  1/9/2020-30/11/2020  1/12/2020-28/2/2021  1/3/2021-30/5/2021  1/6/2021-30/8/2021  1/9/2021-30/11/2021  1/12/2021-28/2/2022  1/3/2022-30/5/2022  1/6/2022-30/8/2022  1/9/2022-30/11/2022  1/12/2022-28/2/2023 | 0  0  2  0  0  0  1  1  20  100  100  39  1 |
|  | USA | Omicron  (BA.5.2.1) | 1/12/2019 – 28/2/2020  1/3/2020- 30/5/2020  1/6/2020- 30/8/2020  1/9/2020-30/11/2020  1/12/2020-28/2/2021  1/3/2021-30/5/2021  1/6/2021-30/8/2021  1/9/2021-30/11/2021  1/12/2021-28/2/2022  1/3/2022-30/5/2022  1/6/2022-30/8/2022  1/9/2022-30/11/2022  1/12/2022-28/2/2023 | 0  0  0  0  0  0  0  0  0  100  100  100  100 |
| 3 | India | Alpha  (B.1.1.7) | 1/12/2019 – 28/2/2020  1/3/2020- 30/5/2020  1/6/2020- 30/8/2020  1/9/2020-30/11/2020  1/12/2020-28/2/2021  1/3/2021-30/5/2021  1/6/2021-30/8/2021  1/9/2021-30/11/2021  1/12/2021-28/2/2022  1/3/2022-30/5/2022  1/6/2022-30/8/2022  1/9/2022-30/11/2022  1/12/2022-28/2/2023 | 0  0  0  1  24  32  0  0  0  0  0  0  0 |
|  | India | Beta  (B.1.351) | 1/12/2019 – 28/2/2020  1/3/2020- 30/5/2020  1/6/2020- 30/8/2020  1/9/2020-30/11/2020  1/12/2020-28/2/2021  1/3/2021-30/5/2021  1/6/2021-30/8/2021  1/9/2021-30/11/2021  1/12/2021-28/2/2022  1/3/2022-30/5/2022  1/6/2022-30/8/2022  1/9/2022-30/11/2022  1/12/2022-28/2/2023 | 0  0  0  0  5  3  0  0  0  0  0  0  0 |
|  | India | Delta  (B.1.617.2) | 1/12/2019 – 28/2/2020  1/3/2020- 30/5/2020  1/6/2020- 30/8/2020  1/9/2020-30/11/2020  1/12/2020-28/2/2021  1/3/2021-30/5/2021  1/6/2021-30/8/2021  1/9/2021-30/11/2021  1/12/2021-28/2/2022  1/3/2022-30/5/2022  1/6/2022-30/8/2022  1/9/2022-30/11/2022  1/12/2022-28/2/2023 | 0  0  0  0  0  100  77  100  5  0  0  0  0 |
|  | India | Omicron  (BA.1.1) | 1/12/2019 – 28/2/2020  1/3/2020- 30/5/2020  1/6/2020- 30/8/2020  1/9/2020-30/11/2020  1/12/2020-28/2/2021  1/3/2021-30/5/2021  1/6/2021-30/8/2021  1/9/2021-30/11/2021  1/12/2021-28/2/2022  1/3/2022-30/5/2022  1/6/2022-30/8/2022  1/9/2022-30/11/2022  1/12/2022-28/2/2023 | 0  0  0  0  0  0  0  4  6  15  0  0  0 |
| 4 | France | Alpha  (B.1.1.7) | 1/12/2019 – 28/2/2020  1/3/2020- 30/5/2020  1/6/2020- 30/8/2020  1/9/2020-30/11/2020  1/12/2020-28/2/2021  1/3/2021-30/5/2021  1/6/2021-30/8/2021  1/9/2021-30/11/2021  1/12/2021-28/2/2022  1/3/2022-30/5/2022  1/6/2022-30/8/2022  1/9/2022-30/11/2022  1/12/2022-28/2/2023 | 0  3  0  3  200  200  100  11  3  0  0  0  0 |
|  | France | Beta  (B.1.351) | 1/12/2019 – 28/2/2020  1/3/2020- 30/5/2020  1/6/2020- 30/8/2020  1/9/2020-30/11/2020  1/12/2020-28/2/2021  1/3/2021-30/5/2021  1/6/2021-30/8/2021  1/9/2021-30/11/2021  1/12/2021-28/2/2022  1/3/2022-30/5/2022  1/6/2022-30/8/2022  1/9/2022-30/11/2022  1/12/2022-28/2/2023 | 0  0  0  0  17  22  1  0  0  0  0  0  0 |
|  | France | Gamma  (P.1) | 1/12/2019 – 28/2/2020  1/3/2020- 30/5/2020  1/6/2020- 30/8/2020  1/9/2020-30/11/2020  1/12/2020-28/2/2021  1/3/2021-30/5/2021  1/6/2021-30/8/2021  1/9/2021-30/11/2021  1/12/2021-28/2/2022  1/3/2022-30/5/2022  1/6/2022-30/8/2022  1/9/2022-30/11/2022  1/12/2022-28/2/2023 | 0  0  0  0  2  0  15  0  0  0  0  0  0 |
|  | France | Delta  (B.1.617.2) | 1/12/2019 – 28/2/2020  1/3/2020- 30/5/2020  1/6/2020- 30/8/2020  1/9/2020-30/11/2020  1/12/2020-28/2/2021  1/3/2021-30/5/2021  1/6/2021-30/8/2021  1/9/2021-30/11/2021  1/12/2021-28/2/2022  1/3/2022-30/5/2022  1/6/2022-30/8/2022  1/9/2022-30/11/2022  1/12/2022-28/2/2023 | 0  0  0  0  0  1  100  100  89  0  0  0  0 |
|  | France | Omicron  (BA.1.1) | 1/12/2019 – 28/2/2020  1/3/2020- 30/5/2020  1/6/2020- 30/8/2020  1/9/2020-30/11/2020  1/12/2020-28/2/2021  1/3/2021-30/5/2021  1/6/2021-30/8/2021  1/9/2021-30/11/2021  1/12/2021-28/2/2022  1/3/2022-30/5/2022  1/6/2022-30/8/2022  1/9/2022-30/11/2022  1/12/2022-28/2/2023 | 0  0  0  0  0  0  0  0  100  29  0  0  0 |
|  | France | Omicron  (BA.5.2.1) | 1/12/2019 – 28/2/2020  1/3/2020- 30/5/2020  1/6/2020- 30/8/2020  1/9/2020-30/11/2020  1/12/2020-28/2/2021  1/3/2021-30/5/2021  1/6/2021-30/8/2021  1/9/2021-30/11/2021  1/12/2021-28/2/2022  1/3/2022-30/5/2022  1/6/2022-30/8/2022  1/9/2022-30/11/2022  1/12/2022-28/2/2023 | 0  0  0  0  0  0  0  0  0  0  1  0  0 |
| 5. | Germany | Alpha  (B.1.1.7) | 1/12/2019 – 28/2/2020  1/3/2020- 30/5/2020  1/6/2020- 30/8/2020  1/9/2020-30/11/2020  1/12/2020-28/2/2021  1/3/2021-30/5/2021  1/6/2021-30/8/2021  1/9/2021-30/11/2021  1/12/2021-28/2/2022  1/3/2022-30/5/2022  1/6/2022-30/8/2022  1/9/2022-30/11/2022  1/12/2022-28/2/2023 | 0  0  0  0  2  1  0  0  0  0  0  0  0 |
|  | Germany | Beta  (B.1.351) | 1/12/2019 – 28/2/2020  1/3/2020- 30/5/2020  1/6/2020- 30/8/2020  1/9/2020-30/11/2020  1/12/2020-28/2/2021  1/3/2021-30/5/2021  1/6/2021-30/8/2021  1/9/2021-30/11/2021  1/12/2021-28/2/2022  1/3/2022-30/5/2022  1/6/2022-30/8/2022  1/9/2022-30/11/2022  1/12/2022-28/2/2023 | 0  0  0  0  3  4  0  0  0  0  0  0  0 |
|  | Germany | Gamma  (P.1) | 1/12/2019 – 28/2/2020  1/3/2020- 30/5/2020  1/6/2020- 30/8/2020  1/9/2020-30/11/2020  1/12/2020-28/2/2021  1/3/2021-30/5/2021  1/6/2021-30/8/2021  1/9/2021-30/11/2021  1/12/2021-28/2/2022  1/3/2022-30/5/2022  1/6/2022-30/8/2022  1/9/2022-30/11/2022  1/12/2022-28/2/2023 | 0  0  0  0  1  2  2  0  0  0  0  0  0 |
|  | Germany | Delta  (B.1.617.2) | 1/12/2019 – 28/2/2020  1/3/2020- 30/5/2020  1/6/2020- 30/8/2020  1/9/2020-30/11/2020  1/12/2020-28/2/2021  1/3/2021-30/5/2021  1/6/2021-30/8/2021  1/9/2021-30/11/2021  1/12/2021-28/2/2022  1/3/2022-30/5/2022  1/6/2022-30/8/2022  1/9/2022-30/11/2022  1/12/2022-28/2/2023 | 0  0  0  0  0  7  22  16  0  0  0  0  0 |
|  | Germany | Omicron  (BA.1.1) | 1/12/2019 – 28/2/2020  1/3/2020- 30/5/2020  1/6/2020- 30/8/2020  1/9/2020-30/11/2020  1/12/2020-28/2/2021  1/3/2021-30/5/2021  1/6/2021-30/8/2021  1/9/2021-30/11/2021  1/12/2021-28/2/2022  1/3/2022-30/5/2022  1/6/2022-30/8/2022  1/9/2022-30/11/2022  1/12/2022-28/2/2023 | 0  0  0  0  0  0  0  0  0  6  0  0  0 |
|  | Germany | Omicron  (BA.5.2.1) | 1/12/2019 – 28/2/2020  1/3/2020- 30/5/2020  1/6/2020- 30/8/2020  1/9/2020-30/11/2020  1/12/2020-28/2/2021  1/3/2021-30/5/2021  1/6/2021-30/8/2021  1/9/2021-30/11/2021  1/12/2021-28/2/2022  1/3/2022-30/5/2022  1/6/2022-30/8/2022  1/9/2022-30/11/2022  1/12/2022-28/2/2023 | 0  0  0  0  0  0  0  0  0  0  0  3  0 |
| 6. | Brazil | Alpha  (B.1.1.7) | 1/12/2019 – 28/2/2020  1/3/2020- 30/5/2020  1/6/2020- 30/8/2020  1/9/2020-30/11/2020  1/12/2020-28/2/2021  1/3/2021-30/5/2021  1/6/2021-30/8/2021  1/9/2021-30/11/2021  1/12/2021-28/2/2022  1/3/2022-30/5/2022  1/6/2022-30/8/2022  1/9/2022-30/11/2022  1/12/2022-28/2/2023 | 0  0  0  0  0  3  0  0  0  0  0  0  0 |
|  | Brazil | Gamma  (P.1) | 1/12/2019 – 28/2/2020  1/3/2020- 30/5/2020  1/6/2020- 30/8/2020  1/9/2020-30/11/2020  1/12/2020-28/2/2021  1/3/2021-30/5/2021  1/6/2021-30/8/2021  1/9/2021-30/11/2021  1/12/2021-28/2/2022  1/3/2022-30/5/2022  1/6/2022-30/8/2022  1/9/2022-30/11/2022  1/12/2022-28/2/2023 | 0  0  1  1  0  3  100  100  21  2  0  0  0 |
|  | Brazil | Delta  (B.1.617.2) | 1/12/2019 – 28/2/2020  1/3/2020- 30/5/2020  1/6/2020- 30/8/2020  1/9/2020-30/11/2020  1/12/2020-28/2/2021  1/3/2021-30/5/2021  1/6/2021-30/8/2021  1/9/2021-30/11/2021  1/12/2021-28/2/2022  1/3/2022-30/5/2022  1/6/2022-30/8/2022  1/9/2022-30/11/2022  1/12/2022-28/2/2023 | 0  0  0  0  0  0  8  7  0  0  0  0  0 |
|  | Brazil | Omicron  (BA.1.1) | 1/12/2019 – 28/2/2020  1/3/2020- 30/5/2020  1/6/2020- 30/8/2020  1/9/2020-30/11/2020  1/12/2020-28/2/2021  1/3/2021-30/5/2021  1/6/2021-30/8/2021  1/9/2021-30/11/2021  1/12/2021-28/2/2022  1/3/2022-30/5/2022  1/6/2022-30/8/2022  1/9/2022-30/11/2022  1/12/2022-28/2/2023 | 0  0  0  0  0  0  0  0  29  9  0  0  0 |

**Figure S1. Multiple sequence alignment of variant of concerns and original Wuhan strain (RBD 319-541 AA of Spike Glycoprotein).**

**
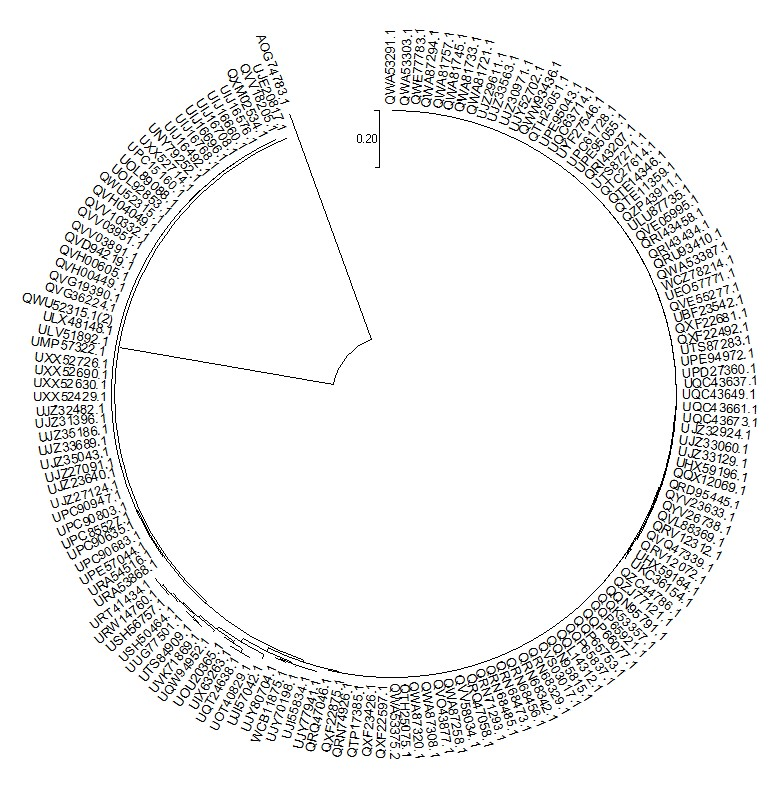
**

**Figure S2. Phylogenetic tree generated for 156 RBD region amino acid sequences of different variants of SARS CoV2 spike glycoprotein involving selected from top five affected countries.**

**Figure S3. Phylogenetic tree for amino acid sequences of RBD region of SARCoV2 variants**

1. (A)

(B)


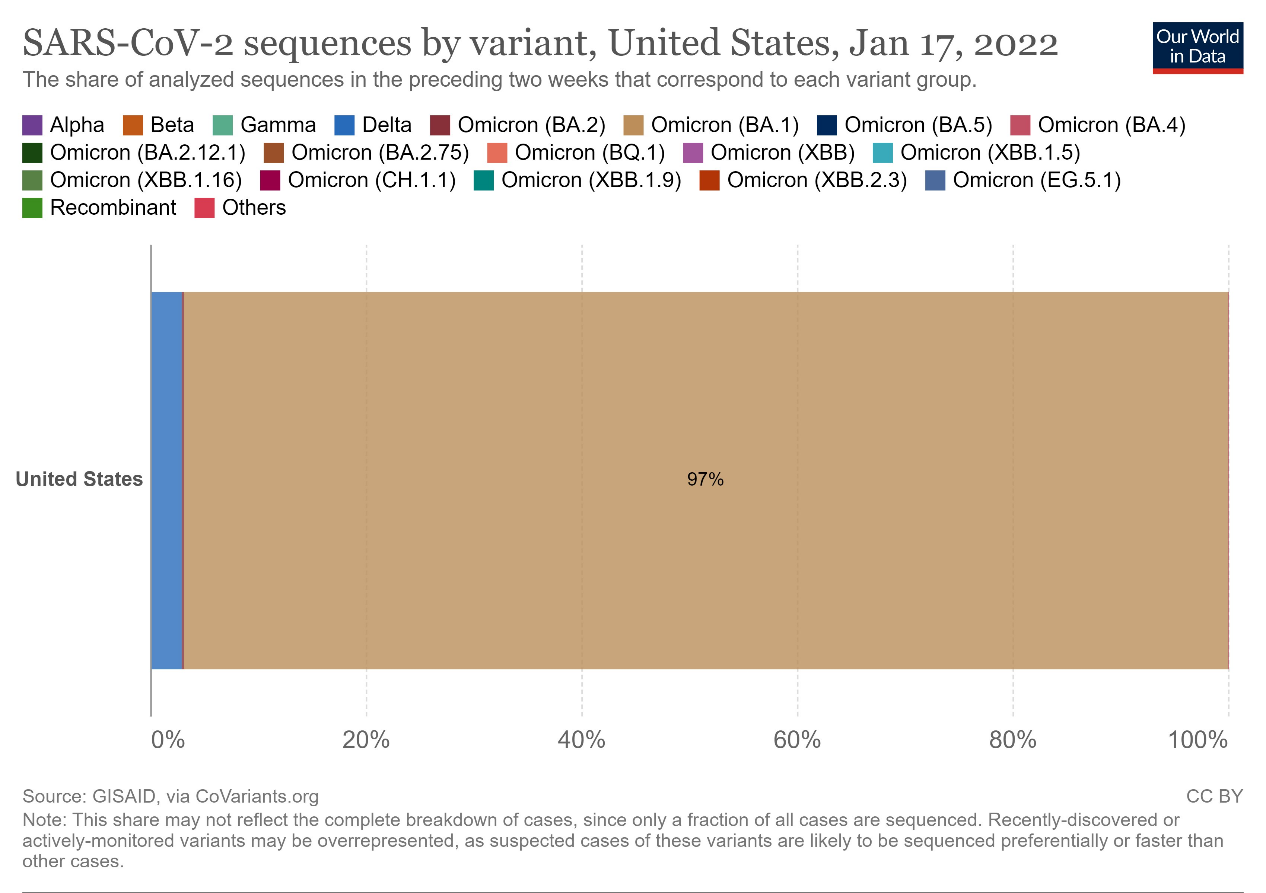


(C)

**Figure S4. (A)-Confirmed new covid-19 cases daily from 1-1-2020 to 1-9-2023 in USA.**

**(B)- Confirmed new covid-19 related deaths daily from 1-1-2020 to 1-9-2023 in USA.**

**(C)- Variants wise sequences analysed during the outbreak of covid-19 in USA.**

**Data has been sourced from WHO COVID-19 Dashboard (https://covid19.who.int).**

1. (A)

(B)

(C)


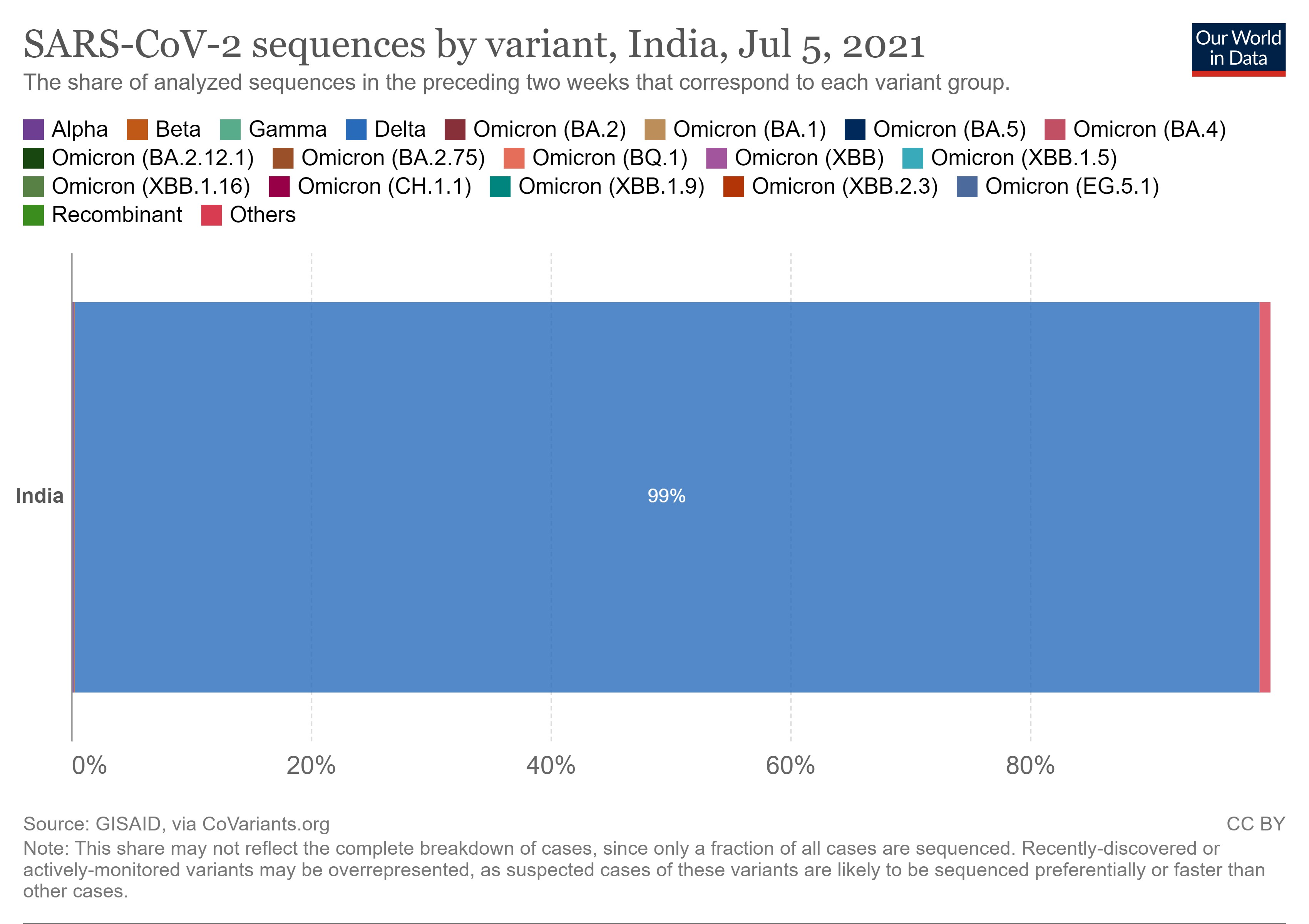


**Figure S5. (A)-Confirmed new covid-19 cases daily from 1-1-2020 to 1-9-2023 in India.**

**(B)- Confirmed new covid-19 related deaths daily from 1-1-2020 to 1-9-2023 in India.**

**(C)- Variants wise sequences analysed during the outbreak of covid-19 in India.**

**Data has been sourced from WHO COVID-19 Dashboard (https://covid19.who.int).**

1. (A)

(B)

(C)


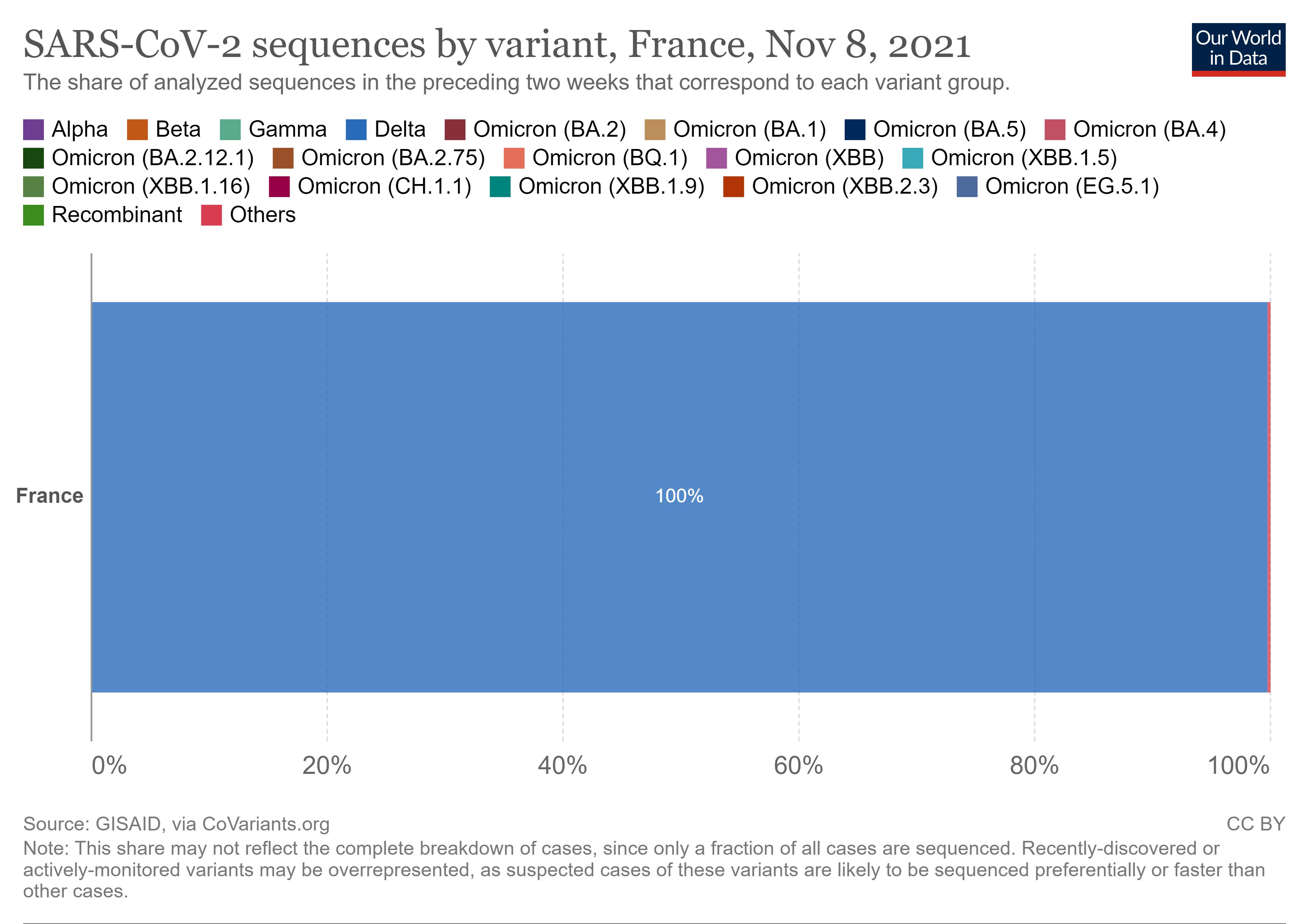


**Figure S6. (A)-Confirmed new covid-19 cases daily from 1-1-2020 to 1-9-2023 in France.**

**(B)- Confirmed new covid-19 related deaths daily from 1-1-2020 to 1-9-2023 in France.**

**(C)- Variants wise sequences analysed during the outbreak of covid-19 in France.**

**Data has been sourced from WHO COVID-19 Dashboard (https://covid19.who.int).**

1. (A)

(B)

(C)


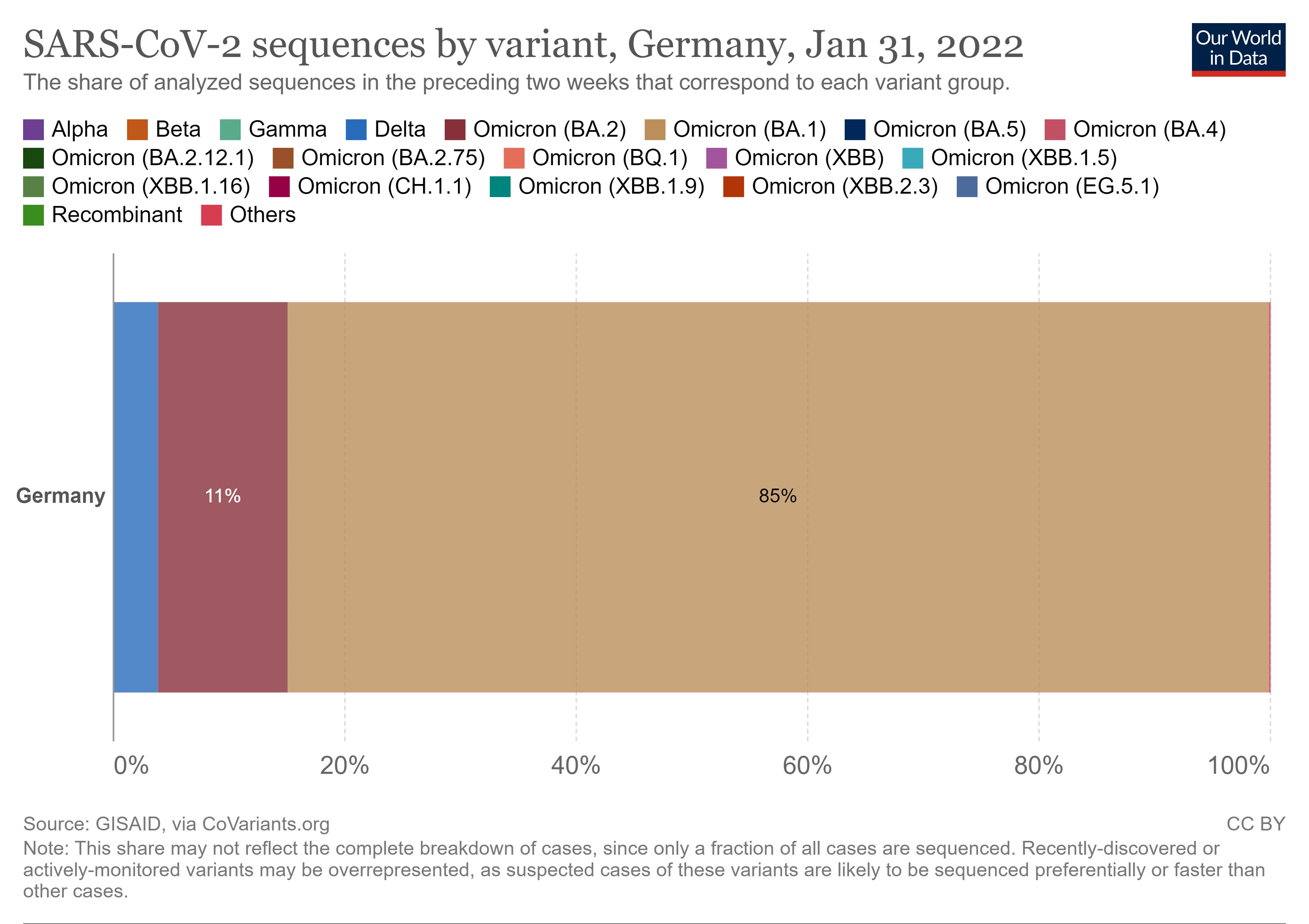


**Figure S7. (A)-Confirmed new covid-19 cases daily from 1-1-2020 to 1-9-2023 in Germany.**

**(B)- Confirmed new covid-19 related deaths daily from 1-1-2020 to 1-9-2023 in Germany.**

**(C)- Variants wise sequences analysed during the outbreak of covid-19 in Germany.**

**Data has been sourced from WHO COVID-19 Dashboard (https://covid19.who.int).**

1. (A)

(B)

(C)


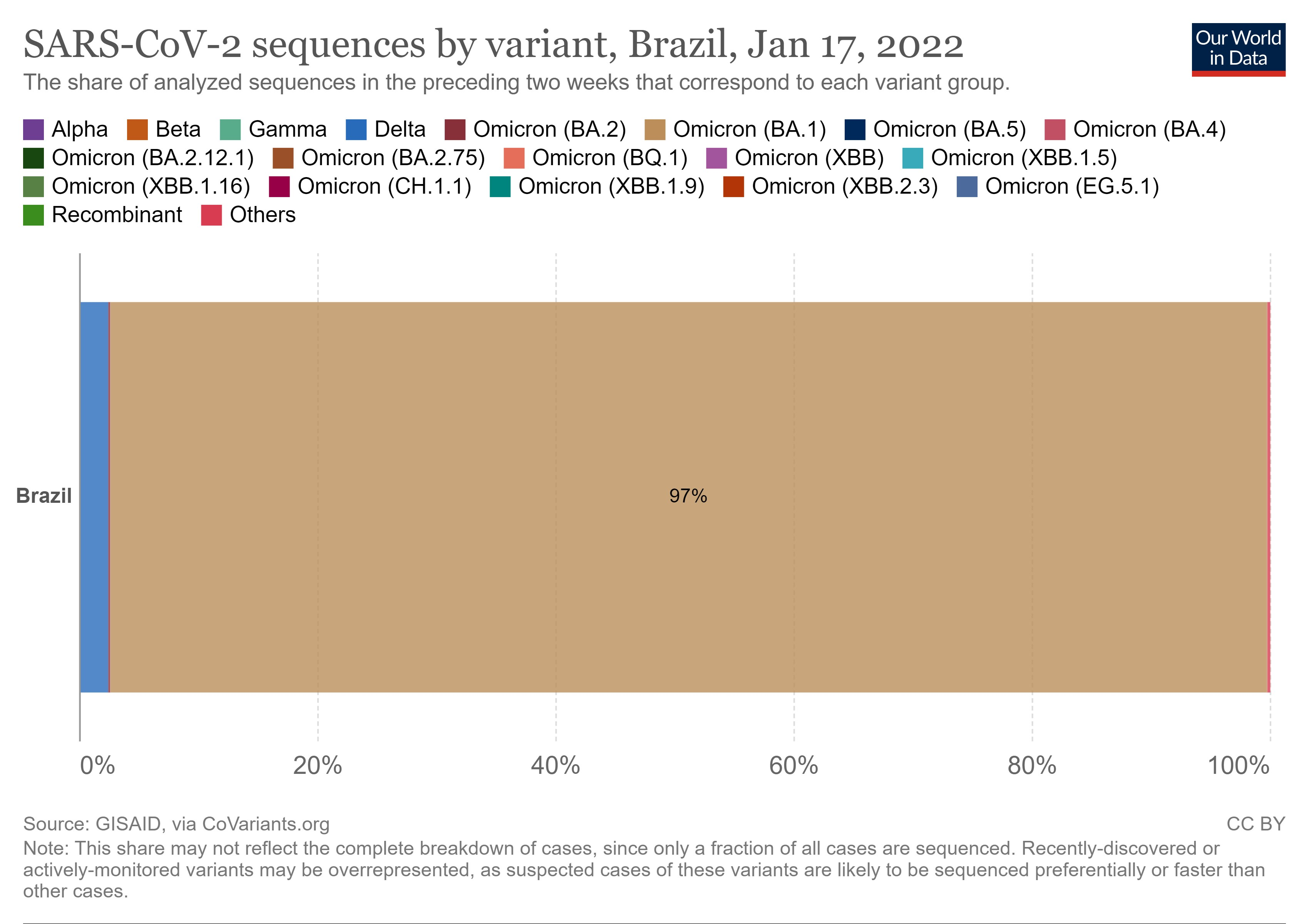


**Figure S8. (A)-Confirmed new covid-19 cases daily from 1-1-2020 to 1-9-2023 in Brazil.**

**(B)- Confirmed new covid-19 related deaths daily from 1-1-2020 to 1-9-2023 in Brazil.**

**(C)- Variants wise sequences analysed during the outbreak of covid-19 in Brazil.**

**Data has been sourced from WHO COVID-19 Dashboard (https://covid19.who.int).**
